# Healthy behaviors and gestational diabetes mellitus in an Iranian setting: A cross-sectional study

**DOI:** 10.1097/MD.0000000000036431

**Published:** 2024-03-01

**Authors:** Maryam Zare, Afrouz Mardi, Paria Yeghanenia, Daniel Hackett

**Affiliations:** a Department of Nutrition, Khalkhal University of Medical Sciences, Khalkhal, Iran; b Department of Public Health, School of Health, Ardabil University of Medical Science, Ardabil, Iran; c Medical Faculty, Ardabil University of Medical Science, Ardabil, Iran; d Physical Activity, Lifestyle, Ageing and Wellbeing Faculty Research Group, School of Health Sciences, Faculty of Medicine and Health, The University of Sydney, Lidcombe, NSW, Australia.

**Keywords:** gestational diabetes mellitus (GDM), healthy behaviors, lifestyle, mothers, nurses

## Abstract

The objective of this study is to determine the healthy behaviors of mothers with gestational diabetes mellitus (GDM) in Ardabil in 2020. An analytic cross-sectional study was conducted on 360 mothers with GDM in Ardabil in 2020. Self-reported data was collected using a sociodemographic and a lifestyle questionnaire (LSQ) with assistance from health staff. Univariate and multivariate linear regression tests were used to assess risk variables associated with health behaviors and GDM. The total score of the LSQ was 123.6 ± 9.9, which was considered fair or average. The participants’ age had a significant relationship with physical health (beta = −.093, *P* = .004), weight control and nutrition (beta = .094, *P* = .010), and psychological health (beta = .081, *P* = .005). Higher educational level was associated with accident prevention (beta = .453, *P* = .001). Participants’ occupation had a significant positive relationship with the prevention of diseases (beta = .925, *P* = .003). A gravida of the participants was associated with weight control and nutrition (beta = −.497, *P* = .016). Body mass index was associated with physical health (beta = −.179, *P* = .001), exercise and fitness (beta = −.149, *P* = .016), psychological health (beta = −.158, *P* = .001), accident prevention (beta = .098, *P* = .023), and total score of LSQ (beta = −.559, *P* = .006). The findings of this study showed that mothers with GDM had LSQ subscales scores considered fair or average, except for the prevention of diseases and avoidance of drugs, alcohol, and opiates subscales, which were considered good.

## 1. Introduction

Gestational diabetes mellitus (GDM) is defined as glucose intolerance in the second or third trimester of pregnancy.^[1]^ Pregnant women gradually develop insulin resistance during pregnancy.^[2]^ Pregnant women’s pancreatic cells may frequently create more insulin to counteract pregnancy hormones in the blood. When the pancreas fails to produce insulin, blood glucose levels rise, leading to gestational diabetes.^[3]^ The prevalence of GDM varies widely from 1% to 45% of total pregnancies in the world.^[4]^ In general, 21.3 million pregnancies are associated with hyperglycemia, and 18.4 million pregnancies are attributed to GDM.^[5]^ In Iran, the prevalence of GDM varied from 1.3% to 18.6% in 2015.^[6]^ However, according to a recent study in 2021, the prevalence of GDM in Iran is 4%.^[7]^ The maternal age, family history of diabetes, excess weight gain in pregnancy, and maternal body mass index (BMI) are the main risk factors for developing GDM.^[8]^ GDM pathophysiology is linked to pregnancy since it alters maternal metabolism to support the fetus’s development.^[9]^ GDM is associated with complications for both the fetus and mother,^[10]^ including gestational hypertensive diseases, preeclampsia, increased cesarean section, a disorder in blood lipid profiles, abdominal obesity, pyelonephritis, preterm labor, and long-term hospitalization,^[11]^ spontaneous abortion, intra uterine growth retardation, fetal death, infant hypoglycemia, malformation, hypoxia, hyperbilirubinemia, cardiac hypertrophy, hypocalcemia, polycythemia, and obesity.^[12,13]^ Some new diagnostic criteria explain, but not all, of the recent rise in prevalence.^[14]^ Based on the International Association of Diabetes and Pregnancy Study Groups, GDM is confirmed with a 1-step screening approach (currently preferred by the American Diabetes Association), and a 2-step Carpenter-Coustan screening approach (recommended by the American College of Obstetricians and Gynecologists). These different approaches have led to the careful reconsideration of the diagnostic criteria for GDM.^[15,16]^

The socioeconomic status, inactivity, obesity, Asian race, and smoking are associated with gestational diabetes.^[17]^ Seven low-risk factors mentioned in diabetes management guidelines include no current smoking, moderate alcohol consumption, regular physical activity, healthy diet, adequate sleeping, and appropriate social relationship.^[18]^ Habitual daily behaviors of people have an effect on health, quality of life, and prevention of diseases. Adopting a health-promoting lifestyle is a safe way to maintain and promote maternal and infant health. This strategy includes performing appropriate health behaviors in various aspects of life that lead to improved physical and mental health and prevention of chronic diseases.^[19]^ Therefore, adherence to healthy behaviors throughout pregnancy and diabetes may enhance pregnancy outcomes and mother and child health.^[20,21]^ The obesity and excessive pregnancy weight gain also increases the risk of GDM.^[22]^ Physical activity during pregnancy is associated with improved psychological status and reduced risk of GDM.^[23]^ Adherence to a healthy dietary pattern before pregnancy is associated with decreased risk of GDM.^[24]^

In the past few decades, low and middle-income countries have undergone rapid industrialization, urbanization, economic development, and market globalization. This development has led to many changes in health outcomes and behaviors.^[25]^ Numerous cross-sectional or interventional studies have focused on GDM and related issues including counseling, diet, BMI, and exercise in Iran.^[26,27]^ However, there is little information in the literature regarding behaviors of women with GDM in Ardabil. The behaviors changes are needed to promote healthy lifestyles in this population. Therefore, this study aimed to determine the healthy behaviors of mothers with GDM in Ardabil.

## 2. Methods

### 2.1. Design, setting, and sample

This analytic cross-sectional study was conducted as a multicenter study on mothers with GDM who attended Ardabil health care centers. Ardabil city is located in the northwest of Iran; 10 health care centers were selected from the 5 regions of the city by stratified cluster sampling. In each center, first the number of eligible mothers was established, and then a random selection was made (through draws) based on the population rates of the centers. The sample size required for the present study was 360 participants based on the equation and the following formula: *P* = .04, significant level was 5% (α = .05) and *d* = .04.^[7]^


$$\begin{array}{l} n=\frac{Z_{1-(\alpha /2)}^{2}p(1-p)}{d^{2}} \end{array}$$

### 2.2. Inclusion and exclusion criteria

People were eligible for inclusion in the study if they were mothers with GDM during 24 to 28 weeks of gestational age, willing to participate, and literate. Exclusion criteria included having pregestational diabetes, and suffering from any chronic disease, and following specific diets or being vegetarian, and women with history of GDM. Data collection was done in 2020.

### 2.3. Procedure and measures

Demographic and medical history characteristics (age, education level, employment status, number of children, gravida, history of stillbirth, BMI, etc) were obtained based on data submitted in the Integrated Health Record System (SIB) of the National Health Service Iran by health providers.

Healthy behaviors data were obtained by a lifestyle questionnaire (LSQ), developed by Lali et al.^[28]^ The LSQ involves 70 questions and measures 10 factors, including physical health, exercise and fitness, weight control and nutrition, prevention of diseases, psychological health, spiritual health, social health, avoidance of drugs, alcohol, and opiates, accident prevention, and environmental health. The items of this questionnaire are scored on a 4-point Likert scale (never = 0, sometimes = 1, usually = 2, always = 3). The score of each scale is obtained by summing up the total scores of the questions for each dimension. This questionnaire has a maximum score of 210 and a minimum score of zero. A healthy lifestyle is defined as a score of about 210, whereas an unhealthy lifestyle is defined as a score of around 70. This questionnaire was completed by the health staff and all information was self-reported by the participants. The questionnaire was validated based on content, face, and concept validity.^[28]^ The scores of the LSQ subscales was categorized as follows: physical health: poor (0–8), fair or average (8.1–16), and good (16.1–24); exercise and fitness: poor (0–7), fair or average (7.1–14), and good (14.1–21); weight control and nutrition: poor (0–7), fair or average (7.1–14), and good (14.1–21); prevention of diseases: poor (0–7), fair or average (7.1–14), and good (14.1–21); psychological health: poor (0–7), fair or average (7.1–14), and good (14.1–21); spiritual health: poor (0–6), fair or average (6.1–12), and good (12.1–18); social health: poor (0–7), fair or average (7.1–14), and good (14.1–21); avoidance of drugs, alcohol, and opiates: poor (0–6), fair or average (6.1–12), and good (12.1–18); accident prevention: poor (0–8), fair or average (8.1–16), and good (16.1–24); and environmental health: poor (0–7), fair or average (7.1–14), and good (14.1–21). This scoring range was divided into poor (0–70), fair or average (70.1–140), and good (140.1–210) categories.

### 2.4. Statistical analysis

Data were examined using statistical software (SPSS version 20). The frequency and percentage of categorical variables, and the mean and standard deviation (SD) of all continuous variables were calculated. Univariate and multivariate linear regression tests were used to examine sociodemographic factors associated with healthy behaviors in mothers with GDM. The Kolmogorov-Smirnov test was used to determine normality of the data, with the results indicating that data was normally distributed. The significance criterion was set at *P* ≤ .05.

The Ethical Committee of Ardabil University of Medical Sciences approved the study (IR.ARUMS.REC.1399.400).

## 3. Results

### 3.1. Sociodemographic characteristics

A total of 360 mothers completed the questionnaires. Table 1 presents the participants’ sociodemographic characteristics. The mean (SD) age of participants was 29.9 (4.4) years and the age of most mothers (38.4%) ranged from 26 to 30 years. The majority of participants had diploma and primary education (70.5%) and were housewives (85%). Most of the mothers were overweight (66.3%), and had a history of 2 pregnancies (44%).

**Table 1:** Sociodemographic and medical history characteristics in mothers with gestational diabetes mellitus.

Characteristic		Participants
Age, yr, mean (SD)		29.9 (4.4)
		Frequency (%)
	18–25	69 (19.2)
	26–30	138 (38.4)
	31–35	112 (31.2)
	36–40	40 (11.1)
Education level	Diploma & primary	253 (70.5)
	University	106 (29.5)
Occupation	Housewives	305 (85)
	Employed	54 (15)
BMI (kg/m^2^)	Normal	78 (21.7)
	Overweight	238 (66.3)
	Obesity	43 (12)
Gravida	1	134 (37.3)
	2	158 (44)
	3	58 (16.2)
	4	9 (2.5)
History of stillbirth	Yes	29 (8.1)
	No	330 (91.9)
History of abnormal infant	Yes	5 (4.4)
	No	354 (98.6)
History of having an infant over 4500 g of weight	Yes	26 (7.2)
	No	333 (92.8)
History of infant death	Yes	7 (1.9)
	No	352 (98.1)

Values are expressed as mean—standard deviation or percentage.

BMI = body mass index, SD = standard deviation.

### 3.2. Healthy behaviors

The mean LSQ subscale scores included 13.1 ± 2.6 for physical health that this score was fair or average, 8.7 ± 3 for exercise and fitness that this score was fair or average, 10.4 ± 2.9 for weight control and nutrition that this score was fair or average, 15 ± 2.1 for prevention of diseases that this score was good, 11.0 ± 2.3 for psychological health that this score was fair or average, 9.7 ± 2.2 for spiritual health that this score was fair or average, 12.1 ± 2.4 for social health that this score was fair or average, 16.8 ± 0.7 for avoidance of drugs, alcohol, and opiates that this score was good, 13.8 ± 2.1 for accident prevention that this score was fair or average, and 13.1 ± 1.9 for environmental health. The total score of LSQ was 123.6 ± 9.9, which was considered fair or average. The highest scores were obtained for the avoidance of drugs, alcohol, and opiates, followed by the prevention of diseases scale, and the lowest scores were obtained for exercise and fitness, weight control and nutrition, and psychological health, respectively (Table 2). The scores of the LSQ subscales were fair or average, except for the prevention of diseases and avoidance of drugs, alcohol, and opiates subscales which were considered good.

**Table 2:** The mean scores of lifestyle questionnaire in mothers with gestational diabetes mellitus.

Scale	Items	Scope range in the research population	The range of possible scores in the questionnaire	Population scores
				Mean (SD)
Physical Health	8	0–24	3–20	13.1 (2.6)
Exercise and Fitness	7	0–21	1–18	8.7 (3)
Weight Control and Nutrition	7	0–21	1–18	10.4 (2.9)
Prevention of Diseases	7	0–21	9–20	15 (2.1)
Psychological Health	7	0–21	4–20	11.0 (2.3)
Spiritual Health	6	0–18	3–15	9.7 (2.2)
Social Health	7	0–21	6–20	12.1 (2.4)
Avoidance of Drugs, Alcohol, and Opiates	6	0–18	15–18	16.8 (0.7)
Accident Prevention	8	0–24	7–21	13.8 (2.1)
Environmental Health	7	0–21	6–18	13.1 (1.9)
Total Score of Lifestyle Questionnaire	70	0–210	75–151	123.6 (9.9)

Values are expressed as mean—standard deviation.

SD = standard deviation.

Univariate linear regression showed that increased age was associated with reduced physical health (beta = −.125, *P* < .001). Higher educational level of women with GDM associated with better accident prevention (beta = .510, *P* < .001) and total score of LSQ (beta = 1.44, *P* = .023). Occupation was associated with exercise and fitness (beta = .867, *P* = .049), prevention of diseases (beta = 0.906, *P* = .003), accident prevention (beta = .629, *P* = .042), and total score of LSQ (beta = 3.60, *P* = .014). The gravida of participants associated with weight control and nutrition (beta = −.439, *P* = .025). A higher BMI was associated with reduced physical health (beta = −.223, *P* < .001), exercise and fitness (beta = −.156, *P* = .008), psychological health (beta = −.133, *P* = .003), and lower total score of LSQ (beta = −.542, *P* = .006), and to better accident prevention (beta = .115, *P* = .005) (Table 3).

**Table 3:** Simple linear regression of the association between sociodemographic and medical history characteristics and lifestyle questionnaire scores.

	Age Beta (95% CI)	*P*-value	Educational level	*P*-value	Occupation	*P*-value	Gravida	*P*-value	BMI	*P*-value
Physical Health	.125 (−.184 to − .065)	<.001*	.047 (−.283 to .377)	.781	−.303 (−1.06 to .465)	.435	−.301 (−.649 to .047)	.090	−.223 (−.322 to −.123)	<.001*
Exercise and Fitness	−.030 (−1.00 to −.039)	.391	.212 (−.163 to .587)	.267	.867 (.002–1.732)	.049*	−.329 (−.726 to .068)	.104	−.156 (−.271 to −.042)	.008*
Weight Control and Nutrition	.059 (−.008 to .126)	.085	.337 (−.026 to .700)	.069	.356 (−.487 to 1.19)	.405	−.439 (−.823 to −.055)	.025*	−.055 (−.167 to .058)	.338
Prevention of Diseases	−.030 (−.077 to .018)	.223	.077 (−.181 to .335)	.556	.906 (.316–1.496)	.003*	−.013 (−.288 to .261)	.928	−.005 (−.084 to .075)	.911
Psychological Health	.040 (−.014 to .093)	.145	.165 (−.124 to .455)	.262	.177 (−.494 to .847)	.605	−.297 (−.603 to .008)	.057	−.133 (−.221 to −.045)	.003*
Spiritual Health	.033 (−.017 to .084)	.199	.107 (−.166 to .380)	.44	.227 (−.405 to .859)	.481	.057 (−.233 to .346)	.701	−.032 (−.116 to .052)	.458
Social Health	−.029 (−.084 to .026)	.303	−.504 (−.352 to .244)	.721	.283 (−.407 to .973)	.42	.121 (−.195 to .437)	.452	−.021 (−.113 to .071)	.66
Avoidance of Drugs, Alcohol, and Opiates	−.004 (−.020 to .013)	.658	.024 (−.065 to .113)	.599	−.002 (−.208 to .205)	.988	.011 (−.084 to .105)	.824	−.010 (−.038 to .017)	.47
Accident Prevention	.036 (−.013 to .085)	.147	.510 (.252 to .769)	<.001*	.629 (.022–1.23)	.042*	.057 (−.223 to .337)	.689	.115 (.034 to .195)	.005*
Environmental Health	.014 (−.031 to .059)	.551	.020 (−.224 to .264)	.87	.467 (−.096 to 1.03)	.103	−.170 (−.429 to .088)	.195	−.023 (−.099 to .052)	.542
Total Score of Lifestyle Questionnaire	−.036 (−.268 to .197)	.762	1.44 (.200–2.69)	.023*	3.60 (.726–6.48)	.014*	−1.30 (−2.62 to .020)	.054	−.542 (−.925 to −.159)	.006*

BMI = body mass index, CI = confidence interval.

* *P* < .05.

Multivariate linear regression indicated that increasing the participants’ age had a significant relationship with the reduction in physical health (beta = −.093, *P* = .004) and the increasing of weight control and nutrition (beta = .094, *P* = .010), and psychological health (beta = .081, *P* = .005). Higher educational level was associated with better accident prevention (beta = .453, *P* = .001). Participants’ jobs had a significant positive relationship with the prevention of diseases (beta = .925, *P* = .003). With increasing gravida, participants were associated with a negative relationship with weight control and nutrition (beta = −.497, *P* = .016). The increased BMI of patients was associated with reduced physical health (beta = −.179, *P* = .001), exercise and fitness (beta = −.149, *P* = .016), psychological health (beta = −.158, *P* = .001), better accident prevention (beta = 0.098, *P* = .023), and lower total score of LSQ (beta = −.559, *P* = .006) (Table 4).

**Table 4:** Multiple linear regression of the association between sociodemographic and medical history characteristics and lifestyle questionnaire scores.

	Age Beta (95%CI)	*p*-value	Educational level	*p*-value	Occupation	*p*-value	Gravida	*p*-value	BMI	*p*-value
Physical Health	−.093 (−.157 to − .030)	.004*	.148 (−.188 to .483)	.387	−.372 (−1.13 to .392)	.339	−.019 (−.378 to .341)	.919	−.179 (−.283 to − .075)	.001*
Exercise and Fitness	.004 (−.071 to .078)	0.924	.126 (−.265 to .517)	0.525	.777 (−.113 to 1.66)	.087	−.205 (−.623 to .214)	.337	−.149 (−.270 to − .028)	.016*
Weight Control and Nutrition	.094 (.022 to .166)	.010*	.244 (−.134 to .622)	0.205	.154 (−.708 to 1.01)	.726	−.497 (−.901 to − .092)	.016*	−.077 (−.195 to .040)	0.194
Prevention of Diseases	−.035 (−.086 to .016)	0.181	.014 (−.283 to .255)−	0.919	.925 (.311–1.538)	.003*	.047 (−.241 to .336)	0.747	.010 (−.074 to .093)	0.816
Psychological Health	.081 (.024 to .137)	.005*	.130 (−.169 to .428)	0.394	.061 (−.619 to .742)	.860	−.299 (−.618 to .021)	.067	−.158 (−.251 to − .066)	.001*
Spiritual Health	.039 (−.015 to .094)	0.157	.096 (−.192 to .384)	0.512	.164 (−.492 to .820)	0.623	.049 (−.259 to .357)	.756	−.056 (−.145 to .033)	0.219
Social Health	−.035 (−.095 to .024)	.244	−.056 (−.371 to .258)	0.724	.338 (−.378 to 1.05)	0.354	.182 (−.155 to .518)	.289	−.012 (−.110 to .085)	0.801
Avoidance of Drugs, Alcohol, and Opiates	−.003 (−.021 to .014)	0.704	.033 (−.061 to .127)	.495	−.019 (−.233 to .196)	0.865	.028 (−.073 to .129)	.588	−.011 (−.040 to .019)	0.474
Accident Prevention	.012 (−.039 to .063)	0.647	.453 (.183 to .724)	.001*	.354 (−.263 to .970)	0.26	.046 (−.244 to .336)	0.755	.098 (.014 to .181)	.023*
Environmental Health	.028 (−.021to .076)	.267	−.064 (−.320 to .193)	0.626	.486 (−.098 to 1.07)	.103	−.200 (−.474 to .075)	0.154	−.024 (−.104to .055)	0.546
Total Score of Lifestyle Questionnaire	.090 (−.155 to .336)	0.469	1.09 (−.198 to 2.39)	0.097	2.86 (−.080 to 5.81)	.057	−.866 (−2.25 to .519)	.220	−.559 (−.960 to − .159)	.006*

CI = confidence interval.

* *P* < .05.

## 4. Discussion

If GDM is not adequately controlled there are many harmful repercussions for mothers and newborns. The controlling of GDM appears to be linked to healthy behaviors. The adjustment of behaviors is emphasized in recent recommendations for the prevention of GDM.^[29]^ The findings from the present study indicate that the LSQ subscales were fair or average, except for the prevention of diseases and avoidance of drugs, alcohol, and opiates subscales which were considered good. There were inverse relationships between age and BMI and physical health, as well as BMI and psychological health, whereas there was a direct relationship between education and accident prevention, as well as occupation with health and fitness and diseases prevention. The increasing gravida had an inverse relationship with weight control and nutrition. An increased BMI was associated with reduced physical health, exercise and fitness, psychological health, and total LSQ score.

In a study by Gharachourlo et al^[30]^ in Iran, the highest behavior score for mothers with GDM was related to environmental health and accident prevention respectively before intervention; these results were in contrast to the present results. Exercise and fitness had the lowest score in their research, and this finding was similar with the current study. Furthermore, the overall score was greater in the previous study than in the present study, suggesting lower levels of healthy behavior in our study. The reason for these differences can be due to the cultural differences between the subjects of the Gharachourlo study and the present study. The participants of the current study are from a region where most of them are Azeri speakers and have a different culture compared to the study of Gharachourlo, and this cultural difference can be the cause of these differences. According to Günther et al (2022), variables such as overall dietary quality rankings, low physical activity, mothers’ smoking, and low mental health, particularly changing the relationship between maternal age and BMI before pregnancy, was not associated with the incidence of GDM. However, vigorous physical activity in early pregnancy was associated with a lower risk of GDM.^[31]^ Günther results were similar to the present results. The results of the present study indicated a significant inverse relationship between maternal age and physical health, between gravida and weight control and nutrition, between BMI and physical and psychological health, exercise and fitness, and total score. This study’s results indicated that mothers with academic education had a more favorable status in terms of accident prevention, and total score of LSQ compared to women with lower education. Furthermore, employee mothers had a more favorable status in terms of exercise and fitness, and accident prevention, prevention of diseases, and total score of LSQ than housewives. Our results were consistent with the findings of a study by Kavehfirouz et al^[32]^ who found a significant difference between healthy behaviors of mothers based on education and employment status; hence, higher education level influenced a mother’s tendency to adopt healthy behaviors. The healthy behaviors in employed mothers were significantly higher than in unemployed mothers. Our finding was consistent with the results of Kordi and Hadizadeh^[33]^ because their results indicated that education level and occupation were important factors for improving women’s lifestyles. The results of other studies also confirmed the present study findings.^[34,35]^

Maternal age, overweight, and obesity before pregnancy are risk factors for glucose intolerance during pregnancy, and their links to GDM have been widely documented.^[31,36]^ These factors were significantly related to physical and psychological health dimensions. Access to more and better educational resources, more specialized studies, and greater life skills seem to be factors explaining why participants with higher education levels had more desired behaviors. It seems that, cultural and ethnical fields were associated with levels of knowledge about GDM. Furthermore, paying attention to culture and increasing knowledge could improve an individuals’ information about GDM.^[34,37]^ Increasing maternal working hours outdoors, BMI, household, number and history of abortion, number of pregnancy and childbirth, and a history of diabetes in the family has been found to significantly increase the odds ratio of developing diabetes.^[38]^ Various factors, such as education level, social position, and stronger self-esteem among employed women, as well as social communication, culture, occupation status, income, and access to particular welfare, may influence women’s health and behaviors.^[32]^ It seems that a control-oriented, systematic, and participation-based approach is useful for designing and implementing health promotion programs for women with GDM.^[39]^

### 4.1. Strengths and limitations

The strengths of the present study include the outcomes of this research and revealed some of the characteristics associated with the healthy behaviors that mothers with GDM face. The participants in the present study lived in a province with the same sociocultural characteristics, where there were no ethnic and cultural differences. Using a questionnaire with appropriate validity and reliability was another strength of this study. Some limitations of the study should also be discussed. First, demographic and behavior data were assessed using a self-reported questionnaire, which might lead to misclassification. Finally, because only the residents of Ardabil were included in the study, it remains unclear whether conclusions can be generalized to other ethnic groups.

### 4.2. Implications

To reduce the incidence of complications in GDM, promotion of healthy behaviors appears to be important. Health professionals and nurses should focus on optimizing physical health, exercise and fitness, weight control and nutrition, prevention of diseases, psychological health, spiritual health, social health, avoidance of drugs, alcohol, and opiates, accident prevention, and environmental health. Nurses can implement healthy programs and training with a participation-based approach for mothers to assist with reducing the prevalence of GDM.

## 5. Conclusion

Based on this study’s results, the healthy behaviors of participants were fair or average for the categories of physical health, exercise and fitness, weight control and nutrition, psychological health, spiritual and social health, accident prevention, and environmental health. The results for the subscales prevention of diseases and avoidance of drugs, alcohol, and opiates were considered good. Age, level of education, occupation, gravida, and BMI were factors that found to influence health and well-being of the participants. The findings support the prioritizing of programs by health system authorities in Iran to educate mothers with and without GDM about healthy behaviors to prevent complications and improve maternal and neonatal health.

## Acknowledgments

We acknowledge the respondents and health care center personnel that collaborated on this study and the Ethics Committee of Ardabil University of Medical Sciences.

## Author contributions

**Conceptualization:** Afrouz Mardi, Paria Yeghanenia.

**Data curation:** Afrouz Mardi, Paria Yeghanenia.

**Formal analysis:** Maryam Zare.

**Investigation:** Maryam Zare, Afrouz Mardi.

**Methodology:** Maryam Zare, Afrouz Mardi.

**Project administration:** Afrouz Mardi.

**Writing – original draft:** Maryam Zare, Afrouz Mardi.

**Writing – review & editing:** Maryam Zare, Daniel Hackett.
